# Increased long-term risk of major adverse cardiovascular events in patients with carbon monoxide poisoning: A population-based study in Taiwan

**DOI:** 10.1371/journal.pone.0176465

**Published:** 2017-04-25

**Authors:** Chung-Shun Wong, Ying-Chin Lin, Li-Chin Sung, Tzu-Ting Chen, Hon-Ping Ma, Yung-Ho Hsu, Shin-Han Tsai, Yuh-Feng Lin, Mei-Yi Wu

**Affiliations:** 1 Department of Emergency Medicine, Taipei Medical University—Shuang Ho Hospital, Taipei, Taiwan; 2 Graduate Institute of Clinical Medicine, College of Medicine, Taipei Medical University, Taipei, Taiwan; 3 Department of Emergency Medicine, School of Medicine, College of Medicine, Taipei Medical University, Taipei, Taiwan; 4 Department of Family Medicine, Taipei Medical University-Shuang Ho Hospital, Taipei, Taiwan; 5 Department of Family Medicine, School of Medicine, College of Medicine, Taipei Medical University, Taiwan; 6 Division of Cardiology, Department of Internal Medicine, Taipei Medical University-Shuang Ho Hospital, Taipei, Taiwan; 7 Institute of Epidemiology and Preventive Medicine, College of Public Health, National Taiwan University, Taipei, Taiwan; 8 Division of Nephrology, Department of Internal Medicine, Taipei Medical University-Shuang Ho Hospital, Taipei, Taiwan; 9 Department of Internal Medicine, School of Medicine, College of Medicine, Taipei Medical University, Taipei, Taiwan; 10 College of Public Health and Nutrition, Taipei Medical University, Taipei, Taiwan; Istituto Di Ricerche Farmacologiche Mario Negri, ITALY

## Abstract

**Background:**

Carbon monoxide (CO) poisoning may cause toxicity to the cardiovascular system. However, the association between CO poisoning and the risk of major adverse cardiovascular events (MACE) remains unestablished. We investigated the incidence of MACE after CO poisoning in Taiwan and evaluated whether CO-poisoned individuals had a higher risk of MACE than did the general population.

**Methods:**

Using Taiwan’s National Health Insurance Research Database (NHIRD) during 2005–2013, a nationwide population-based cohort study was conducted among patients who experienced CO poisoning between 2005 and 2013. CO poisoning was defined according to the International Classification of Diseases, Ninth Revision, Clinical Modification codes. The study cohort comprised patients with CO poisoning between 2005 and 2010 (N = 13,939). Each patient was matched according to age, sex and index date with four randomly selected controls from the comparison cohort (N = 55,756). All patients were followed from the study date until MACE development, death, or the end of 2013. The hazard ratios for MACE were compared between the two cohorts by using Cox proportional hazards regressions analyses.

**Results:**

Incident cases of MACE were identified from the NHIRD. After adjustment for potential confounders, the study cohort was independently associated with a higher MACE risk (adjusted hazard ratio, 2.00; 95% confidence interval, 1.83–2.18).

**Conclusion:**

This population-based cohort study indicated that patients with CO poisoning have a higher risk of MACE than do individuals without CO poisoning.

## Introduction

Carbon monoxide (CO) poisoning is a common cause of toxicological morbidity and mortality. It accounts for 50,000 emergency department (ED) visits and 2700 deaths annually in the United States[1]. CO is a toxic gas. It is colorless, odorless, tasteless, and initially non-irritating, therefore, its detection is complicated. Accidental CO poisoning is commonly associated with faulty or inadequately ventilated gas heating appliances, fires, mining accidents, and automobile exhaust fumes. Moreover, CO present in the smoke produced from burning charcoal can be used for committing suicide[2]. CO adversely affects nearly all organs and tissues, and the high oxygen demand of the cardiovascular and central nervous systems causes them to predominate the acute and delayed clinical features[3]. Cardiovascular manifestations of acute CO poisoning include myocardial dysfunction, ischemia and infarction, arrhythmias and cardiac arrest. Myocardial injury with elevated levels of cardiac biomarkers and changes in diagnostic electrocardiogram was frequently reported in moderate to severe CO poisoning [4–6]. However, based on a review of the literature, only a few case reports have confirmed the effects of CO toxicity on the cardiovascular system. Therefore, we conducted a retrospective cohort study for determining the association between CO poisoning and major adverse cardiovascular events (MACE) including acute coronary syndrome, heart failure, cerebrovascular accident, and malignant dysrhythmia. This study provides data on the incidence of MACE after CO poisoning from a longitudinal health insurance database.

## Materials and methods

### Longitudinal health insurance database

Taiwan’s National Health Insurance program, established in 1995, provides mandatory universal health insurance. In 2007, the program covered nearly 99% (>25 million people) of the residents of Taiwan. The database contains detailed patient information, including data on sex; date of birth; residential or work area; dates of clinical visits; the International Classification of Diseases, Ninth Revision, Clinical Modification (ICD-9-CM) diagnosis codes; prescription details; expenditure amounts; and outcomes at hospital discharge (recovered, died, or transferred). The study was approved from full review after the Joint Institutional Review Board of Taipei Medical University. The study was conducted in accordance with approved guidelines. Informed consent of the study participants was not required because the dataset used in this study consisted of de-identified secondary data from the NHI program.

### Study population

The study cohort comprised all patients who received a diagnosis with CO poisoning (ICD-9-CM 986, E868.8) between January 1, 2005, and December 31, 2010. A history of potential carbon monoxide exposure, such as being exposed to a residential fire or suicide attempt, may suggest poisoning, but the coding diagnosis of CO poisoning is confirmed in the hospital by measuring the levels of carbon monoxide in the blood. The exclusion criterion was MACE diagnosis (acute coronary syndrome: ICD-9 410–410.9, or ICD-9 36.0–36.03, 36.05–36.09, 36.1–36.99, and V45.81; heart failure: ICD-9 428.0–428.10; cerebrovascular accident or stroke: ICD-9 430–432, and 433–437; or malignant dysrhythmia: ICD-9 426.0, 426.12–426.13, 426.51–426.52, 426.54, 427.1, 427.4, 427.41–427.42, and 427.5) before the CO exposure index date. Patients with missing variables, such as birth date and sex, were also excluded from the study. We also identified patients receiving MACE diagnoses in at least three consecutive examinations to ensure the accuracy of diagnosis. The resulting study cohort comprised 13,939 patients with CO poisoning. Each patient was then individually followed up from the index ambulatory visit until MACE development, death, or December 31, 2013.

### Matched control sample

The comparison cohort comprised the remaining patients in Taiwan’s National Health Insurance Research Database (NHIRD) between January 1, 2005 and December 31, 2010. Patients with CO poisoning diagnosed during 2004–2013 were excluded from the comparison cohort. Patients with MACE diagnosed before index date matching were not included in the comparison cohort. In total, 55,756 patients were randomly stratified, and four patients were age (± 1 year-), sex-, and index date-matched with each patient from the study cohort. The index date was defined as the first day of CO poisoning, and the index date of the comparison cohort corresponded with that of the study cohort.

### Study endpoint

Each patient was followed from their entry date until development of MACE, death, or December 31, 2013. MACE diagnoses were confirmed according to the ICD-9 codes in at least three consistent visits. Inpatient and outpatient diagnoses were reviewed to enable adjustment for comorbidities, namely diabetes mellitus (DM), cancer, hypertension (HTN), and hyperlipidemia, as well as the Charlson comorbidity index (CCI). The National Cause of Death Registry contains data on all deaths; we assessed data available until December 2013. We used national identification numbers for linking data of the included patients with those obtained from the aforementioned registry.

### Statistical analysis

We compared the demographic data and comorbidities between the study and comparison cohorts. Differences in demographic characteristics and comorbidities were examined using the Pearson chi-squared test or the *t* test. A Cox proportional hazards regression model was used for comparing hazard ratios (HRs) for MACE between the study and comparison cohorts after adjustment for potential confounders, namely DM, cancer, HTN, hyperlipidemia, and CCI. Moreover, dichotomous variables in the model were assessed for proportionality by using exploratory diagnostic log–log survival plots to meet the proportional hazards assumption. We performed sensitivity analysis excluding patients diagnosed with MACE less than 1–2 years after CO poisoning. The study cohort was also reanalyzed after stratification by age (18–44, 45–64,≥ 65 years), sex, comorbidity status, and CCI (≤1 or >1). Moreover, we used Kaplan–Meier analysis for assessing the proportion of patients without MACE in each cohort. The MACE cumulative incidence curves based on the Kaplan–Meier estimates of the survival function for the study and comparison cohorts were plotted. All analyses were performed using the SAS statistical package (SAS System for Windows, Version 9.3.1, SAS Institute Inc., Cary, NC, USA) or SPSS 20 (IBM Corp. Released 2011. IBM SPSS Statistics for Windows, Version 20.0. Armonk, NY: IBM Corp.). A value of P < 0.05 was considered significant.

## Results

Demographic characteristics and comorbidities among the study and comparison cohorts are presented in Table 1. The study cohort had higher rates of comorbidities before the index date than did the comparison cohort. The study cohort was more likely to develop DM, cancer, HTN, and hyperlipidemia and to have a higher CCI (P < 0.001 for all) than was the comparison cohort (Table 1).

**Table 1:** Demographic and clinical characteristics of the study population

Characteristic	Carbon monoxide (N = 13,939)	Reference group (N = 55,756)
Age, mean (SD), y	38.2 ± 13.2	38.2 ± 13.2
Age group, y		
18–39	8,235 (59.1)	32,940 (59.1)
40–59	4,808 (34.5)	19,232 (34.2)
60–79	759 (5.5)	3,036 (5.5)
≥80	137 (1.0)	548 (1.0)
Female sex	7,136 (51.2)	28,544 (51.2)
Comorbid conditions before the date index		
Diabetes mellitus*	850 (6.1)	2060 (3.7)
Cancer*	463 (3.3)	1,345 (2.4)
Hypertension*	1,504 (10.8)	4,610 (8.3)
Hyperlipidemia*	1,018 (7.3)	3,211 (5.8)
Charlson comorbidity index*		
≤1	12,113 (86.9)	51,822 (92.9)
1–3	1,360 (9.8)	3,237 (5.8)
>3	466 (3.3)	697 (1.3)
Mean (SD)*	0.6 ± 1.3	0.3 ± 0.9
Follow-up year*	5.5 ± 2.3	6.0 ± 1.9

Abbreviations: N, sample size; SD, standard deviation.

*P-value<0.001, carbon monoxide compared with reference group.

Table 2 presents the incidence, crude and adjusted HRs for MACE of the two cohorts. Of all patients in the study cohort, 791 [103.5 per 10,000 person-years] experienced MACE during the 76,396.7 person-year follow-up period. The crude HR for MACE was 2.36 (95% confidence interval [CI], 2.16–2.57), and the adjusted HR was 2.00 (95% CI, 1.83–2.18) after adjustment for DM, cancer, HTN, hyperlipidemia, and the CCI.

**Table 2:** Incidence and adjusted hazard ratios for MACE during the 9-year follow-up period.

	Carbon monoxide	Reference group
MACE present	791	1466
No. of person-years	76396.7	335591.5
Incidence/10,000 person-years	103.5	43.7
Crude hazard ratio	2.36 ^a^ (2.16–2.57)	1.00 (reference)
Adjusted hazard ratio^b^	2.00 ^a^ (1.83–2.18)	1.00 (reference)

Abbreviation: MACE, major adverse cardiovascular events.

Notes: Values in parentheses are 95% confidence intervals.

^a^: P-value<0.001.

^b^: Adjustments were made for diabetes mellitus, cancer, hypertension, hyperlipidemia, and the Charlson comorbidity index.

**Fig 1:**
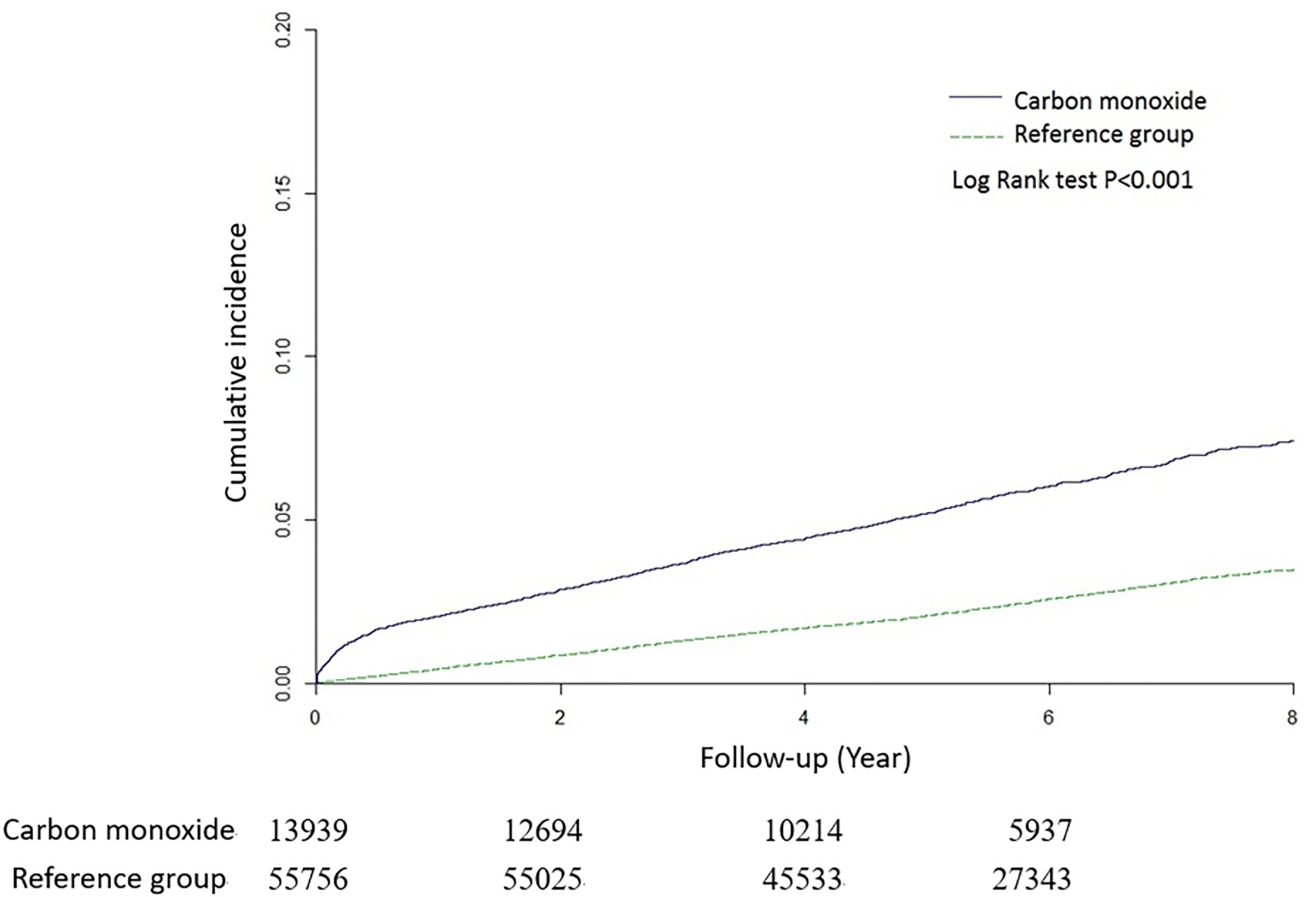
Plot of hazard curves for major adverse cardiovascular events based on the Cox model analysis for patients with carbon monoxide poisoning and controls.

Sensitivity analysis results obtained using a Cox proportional hazards regression model for examining the risk of MACE after CO poisoning are listed in Table 3. We performed sensitivity analysis after excluding patients with MACE diagnosed fewer than 1–2 years after CO poisoning. The association between CO poisoning and MACE remained consistent. The Kaplan–Meier analysis indicated survival curves for the cumulative incidence of MACE in the study and comparison cohorts during the 9-year follow-up period. The cumulative incidence of MACE during the follow-up for the study cohort was significantly higher than that for the comparison cohort (P < 0.001) (Fig 1).

**Table 3:** Sensitivity analysis of the Cox regression model for major adverse cardiovascular events.

	Adjusted HR* (95% CI)	P-value
**Primary analysis**	2.00 (1.83–2.18)	<0.0001
**Excluding patients with event during first year**	1.58 (1.42–1.75)	<0.0001
**Excluding patients with event during first 2 years**	1.58 (1.40–1.77)	<0.0001

Abbreviations: HR, hazard ratio; CI, confidence interval.

*Adjustments were made for diabetes mellitus, cancer, hypertension, hyperlipidemia, and the Charlson comorbidity index.

**Fig 2:**
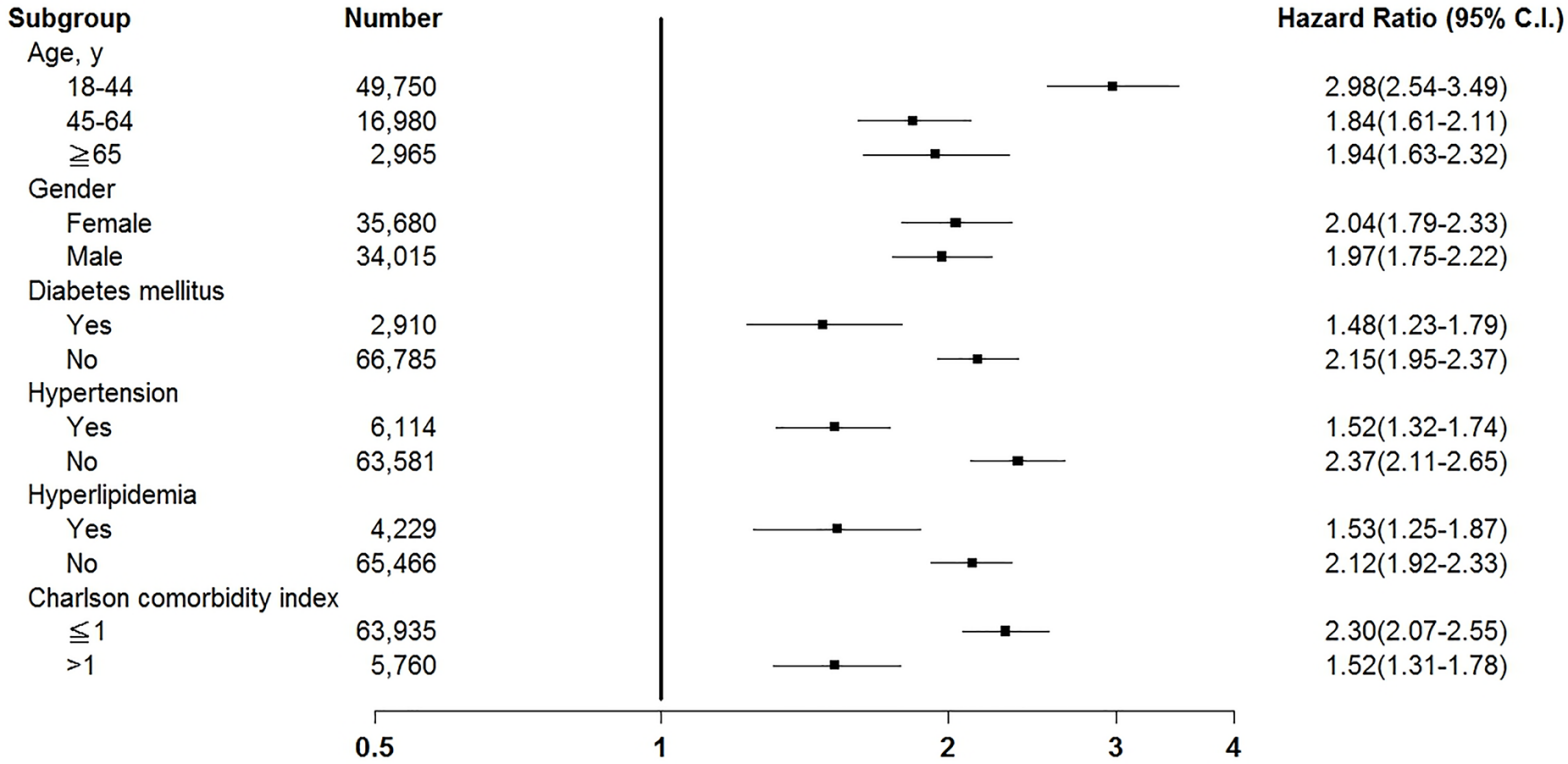
Subgroup analysis of risk of major adverse cardiovascular events in patients with carbon monoxide poisoning and controls.

**Fig 3:**
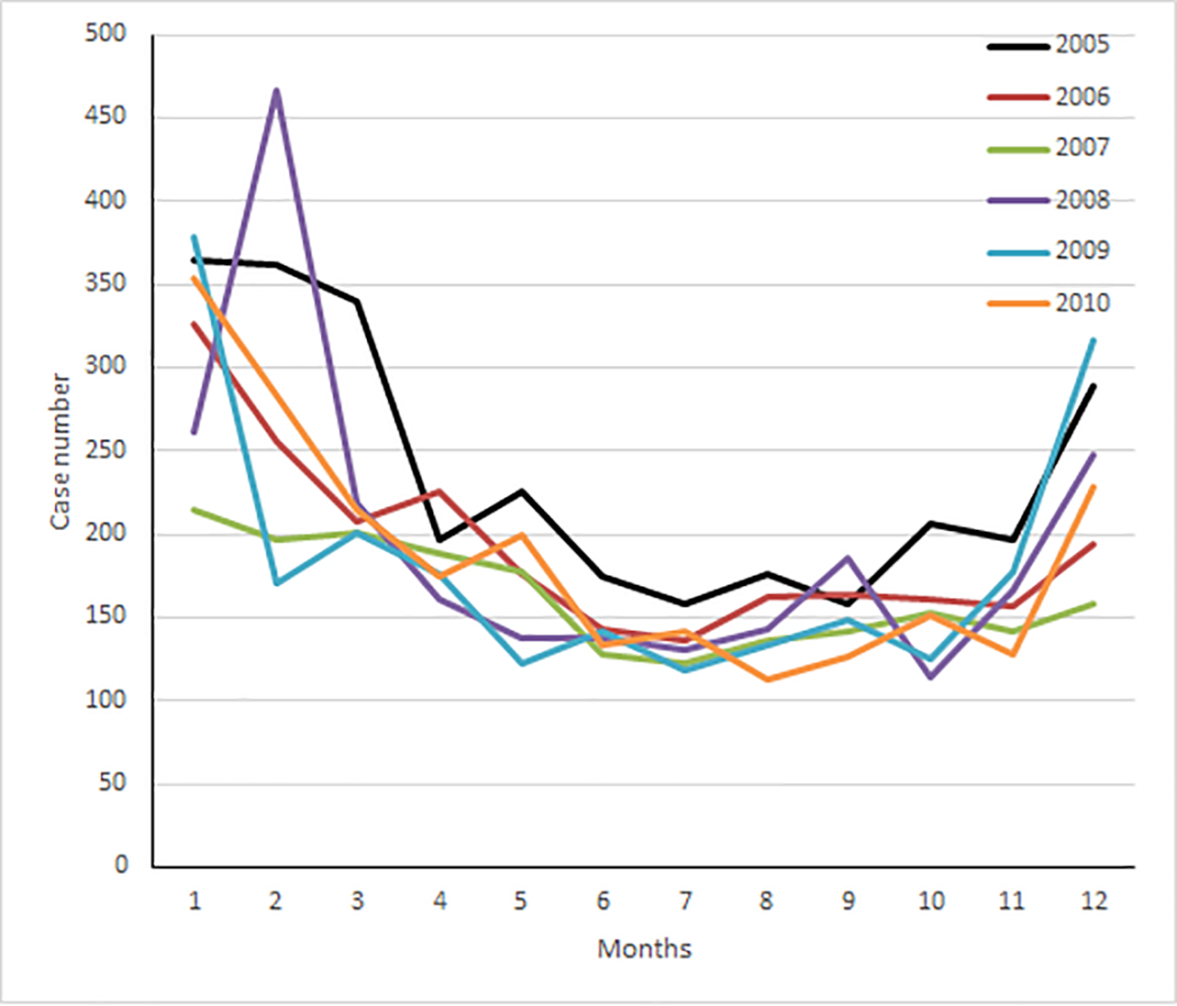
Number of patients with carbon monoxide poisoning during the study period.

After age stratification (Fig 2), the adjusted HR for MACE was 2.98 (95% CI, 2.54–3.49) for patients aged 18–44 years in the study cohort, which was higher than that reported for patients in the comparison cohort. Similarly, the adjusted HRs for MACE were 1.84 (95% CI, 1.61–2.11) and 1.94 (95% CI, 1.63–2.32) in patients aged 45–64 years and ≥ 65 years, respectively, in the study cohort, which were higher than those for patients in the same age groups in the comparison cohort. We analyzed the data stratified by sex, DM, HTN, hyperlipidemia and CCI (≤1 or >1). The adjusted HRs for MACE were higher in the study cohort than in the comparison cohort in all stratifications during the 9-year follow-up period were all higher than that in the comparison cohort, respectively. The distribution of patients who received a diagnosis of CO poisoning by the time of the ED visit is depicted in (Fig 3). The frequency of CO poisoning increased in winter months every year.

## Discussion

CO poisoning remains a major cause of accidental and intentional injuries worldwide. Several studies have indicated that CO poisoning is associated with a risk of toxicity to the central nervous system and heart. The present population-based cohort study conducted using Taiwan’s NHIRD revealed that patients with CO poisoning have a higher risk of MACE than do controls. The study also revealed a significant risk of MACE in a long-term follow-up after adjustment for age, sex, and comorbidity. During multivariable analysis of MACE, the variables of the study and comparison cohorts were balanced by adjustment for DM, cancer, HTN, hyperlipidemia and CCI, thereby eliminating bias. A major strength of this study is the use of a comprehensive national database to analyze the subsequent development of MACE in patients with CO poisoning over a 9-year period. In this population-based study, we demonstrated that CO poisoning is associated with MACE.

The cardiovascular manifestations of CO poisoning have been described only in case reports and entail various types of myocardial dysfunction and injury. Recently, various mechanisms have been proposed. CO exposure reduces the capacity of the blood to carry and deliver oxygen. It also induces marked oxidative stress and inflammatory responses via multiple hypoxia-independent pathways. The direct hypoxic effects, subsequent oxidative stress, and inflammatory responses cause varying degrees of myocardium damage [7, 8]. Other effects, such as relaxation of vessel smooth muscles and autonomic dysfunction caused by systemic hypoxia and compensatory tachyarrhythmia further exacerbates the hypoxic injury of the myocardium [3, 9]. Lee et. al reported that CO poisoning was associated with a 1.83-fold higher risk of arrhythmia compared with the comparison cohort, and non-significantly associated with risk of CAD and CHF [10]. CO poisoning can lead to toxicity of the central nervous system and heart, and death. After acute poisoning, long-term problems may occur. Our study put stress on the occurrence of MACE including cardiovascular and neurology complications. Our findings indicated that the incidence rate of MACE is 103.5 per 10,000 person-years in patients with CO poisoning. We performed a time-trend analysis for evaluating the risk of MACE through stratification based on the event time. The effects of CO poisoning were significant during the follow-up period, implying a long-term MACE risk after CO poisoning.

Elderly patients in the study cohort exhibited a high HR; younger patients also exhibited a considerable risk of MACE after CO exposure, implying that these patients require exclusive clinical care. Some reported cases reveal a close association between cardiac dysfunction and neurologic abnormality [11]. Other studies have demonstrated that myocardial injury independently predicts poor short-term outcomes and long-term mortality in patients with moderate to severe CO poisoning [4, 12]. The standard treatment for CO poisoning includes the administration of oxygen and general supportive care. Some studies have demonstrated that hyperbaric oxygen may be beneficial for severe CO poisoning [13, 14].

Frequent emergency department visits due to CO poisoning in winter months were noted in the literature [15]. The distribution of patients with CO poisoning in the NHIRD, which covers 99% of the residents of Taiwan, also indicated an increase in the frequency of CO poisoning in winter months during 2005–2010. The increase may be related to unintentional poisoning due to the use of natural gas. Implementing public health interventions, such as promoting CO alarm installations, can reduce ongoing exposures to CO from common sources, such as those in the residential setting.

The major strength of our study is the relatively large sizes of the study and comparison cohorts. However, this study has several limitations. First, MACE diagnosis based on ICD-9-CM codes may be less accurate than that obtained according to a complete interview, laboratory data, and clinical information. To validate the MACE diagnoses in this study, we selected only patients who consecutively received diagnoses with MACE by clinical physicians at least three times. However, the study did not consider patients with MACE who did not seek medical care or those patients whose diagnosis was miscoded. Furthermore, for comorbidities, we identified chronic diseases on the basis of patients making at least three visits to the hospital for the same disease. Without conducting a biological examination for disease validation, inaccurate diagnosis is likely, increasing the possibility of bias. Second, data on some variables, such as smoking, body mass index, and laboratory data were not available in the claims database. Although we matched the cohorts to balance their demographics and conducted stratified analysis to confirm the robustness of our results, unmeasured confounders possibly affecting both groups may exist. Prospective studies focusing on measuring these confounders are warranted. This study analyzed data from the Taiwan NHIRD; thus, the generalizability of the findings to populations in other countries is limited.

## Conclusions

In conclusion, CO poisoning is a risk factor for MACE, particularly in young adults, and is independent of known MACE risk factors. The present study provides a basis for understanding the subsequent risk of MACE in patients with CO poisoning and enhances the knowledge of clinicians by identifying evidence of myocardial damage and related heart dysfunction in CO poisoning patients. Further research is required in this area of public health. More emphasis on prevention of CO poisoning, early detection, and HBO therapy is essential for preventing further complications. Furthermore, additional studies are required for elucidating the potential application of these in mitigating the effects of CO poisoning. Public health interventions for the prevention of CO poisoning support positive outcomes.
