# Correction: Increased long-term risk of major adverse cardiovascular events in patients with carbon monoxide poisoning: A population-based study in Taiwan

**DOI:** 10.1371/journal.pone.0215878

**Published:** 2019-04-18

**Authors:** Chung-Shun Wong, Ying-Chin Lin, Li-Chin Sung, Tzu-Ting Chen, Hon-Ping Ma, Yung-Ho Hsu, Shin-Han Tsai, Yuh-Feng Lin, Mei-Yi Wu

There are errors in the author affiliations. The correct affiliations are as follows: Chung-Shun Wong^1,2,3^, Ying-Chin Lin^4,5^, Li-Chin Sung^6,7^, Tzu-Ting Chen^8^, Hon-Ping Ma^1,3^, Yung-Ho Hsu^9,10^, Shin-Han Tsai^1,3,11^, Yuh-Feng Lin^2,9,10^, Mei-Yi Wu^8,9,10^

**1** Department of Emergency Medicine, Taipei Medical University—Shuang Ho Hospital, Taipei, Taiwan, **2** Graduate Institute of Clinical Medicine, College of Medicine, Taipei Medical University, Taipei, Taiwan, **3** Department of Emergency Medicine, School of Medicine, College of Medicine, Taipei Medical University, Taipei, Taiwan, **4** Department of Family Medicine, Taipei Medical University-Shuang Ho Hospital, Taipei, Taiwan, **5** Department of Family Medicine, School of Medicine, College of Medicine, Taipei Medical University, Taiwan, **6** Division of Cardiology, Department of Internal Medicine, Taipei Medical University-Shuang Ho Hospital, Taipei, Taiwan, **7** Division of Cardiology, Department of Internal Medicine, School of Medicine, College of Medicine, Taipei Medical University, Taipei, Taiwan, **8** Institute of Epidemiology and Preventive Medicine, College of Public Health, National Taiwan University, Taipei, Taiwan, **9** Division of Nephrology, Department of Internal Medicine, Taipei Medical University-Shuang Ho Hospital, Taipei, Taiwan, **10** Division of Nephrology, Department of Internal Medicine, School of Medicine, College of Medicine, Taipei Medical University, Taipei, Taiwan, **11** College of Public Health and Nutrition, Taipei Medical University, Taipei, Taiwan
